# Script Voz – clinical case simulator of adults with behavioral dysphonia: planning and content creation

**DOI:** 10.1590/2317-1782/e20240075en

**Published:** 2025-02-03

**Authors:** Lorena Luiza Costa Rosa Nogueira, Sandro Renato Dias, Anna Alice de Figueirêdo Almeida, Renata Rangel Azevedo, Ana Cristina Côrtes Gama

**Affiliations:** 1 Departamento de Fonoaudiologia, Universidade Federal de Minas Gerais – UFMG - Belo Horizonte (MG), Brasil.; 2 Departamento de Computação, Centro Federal de Educação Tecnológica de Minas Gerais – CEFET-MG – Belo Horizonte (MG), Brasil.; 3 Departamento de Fonoaudiologia, Universidade Federal da Paraíba – UFPB- João Pessoa (PB), Brasil.; 4 Departamento de Fonoaudiologia, Universidade Federal de São Paulo – UNIFESP - São Paulo (SP), Brasil.

**Keywords:** Speech Language and Hearing Sciences, Clinical Reasoning, Clinical Decision Making, Cognition, Learning, Simulation Training, Treinamento por Simulação

## Abstract

**Purpose:**

To present the preliminary results of the development of a web platform aimed at training clinical reasoning aimed at the speech therapy approach to patients with behavioral dysphonia.

**Methods:**

Methodological study that describes the tool development stages. The contents were defined by consensus from a panel of experts. The project will comply with the stages of planning, platform development, content creation, usability evaluation and user acceptance, redefinition of the tool and determination of charges. This article presents the processes and results of the first three stages.

**Results:**

Called Script Voz, the web platform will initially include six clinical cases of behavioral dysphonia. The user can select one clinical case at a time, and must be guided sequentially through the assessment, diagnosis, and therapeutic planning stages of the case. To do so, you must choose the answer(s) you deem appropriate to each of the ten questions proposed for each clinical case. Procedure guides for assessment, diagnosis and therapeutic planning focused on clinical cases that will make up the tool are presented, highlighting their processes.

**Conclusion:**

Script Voz will be freely accessible. The procedural guides provided here will guide data collection, manifestation diagnosis and therapeutic planning focused on the clinical cases that will make up the tool. The questions and feedback that will guide the user experience are still in the final stages of preparation. The tool will use real clinical cases and arguments anchored in the clinical reasoning of experienced professionals.

## INTRODUCTION

Clinical thinking in healthcare is the cognitive process that enables professionals to establish the correct diagnosis and the appropriate course of action when faced with a given clinical problem^(1)^. Difficulties in clinical thinking have been reported in most professional fields, especially medicine. A study reveals that the obstacles faced by inexperienced professionals and students concern both the knowledge itself and the strategy in tasks involving clinical decision-making^(2)^.

The National Health Council establishes that the training of speech-language pathologists should provide them with the knowledge required to practice promotion, prevention, and recovery at all levels of care^(3)^. The following skills are worth highlighting: conducting assessments, establishing differential diagnoses, and carrying out therapeutic management, seeking to promote care centered on the needs of users, their families, and the community, in all life cycles^(3)^.

Research has shown that speech therapy undergraduates have difficulties with clinical thinking skills in terms of making diagnoses^(4,5)^. Differences regarding this skill have also been shown to be significant when comparing students with professional speech therapists; the gap relates to better planning of the hierarchy of assessments and better connections between assessment and therapeutic planning by the more experienced students^(5)^.

The literature points to theories that have sought to understand the system for developing clinical thinking over time, starting with the procedural theory. According to this theory^(1)^, when faced with a clinical case, health professionals establish diagnostic hypotheses, which are accepted or refuted based on new information. This theory, however, failed to explain the differences in competence between experienced and beginner professionals, leading to the structural theory^(6)^. The latter, in turn, proposes that clinical thinking relies on acquired knowledge; however, it is not enough to accumulate knowledge, it must be structured in the memory so that it can be used daily, a premise that allowed for the emergence of two types of thinking: analytical and non-analytical^(7)^. The former is used to solve more complex problems, involving the hypothetical-deductive method, while non-analytical thinking is applied to solve more everyday clinical cases, to which the professional has already been repeatedly exposed, having created what the authors called “illness scripts”^(1)^.

Expertise is then gradually established through intense practice. According to this theory, the most critical aspect of learning is deliberate practice, which enriches the repertoire of concepts and improves the storage of problems that have already been solved^(6)^.

Clinical simulation has been used in healthcare as a significant learning strategy, allowing learners to participate actively in the development of their knowledge and providing an alternative for broadening the repertoire of illness scripts^(8)^. Clinical simulation is a technique or technology that seeks to recreate the specificities of real-life situations, enabling the acquisition of skills and competencies in a safe environment, before direct contact with patients^(8)^.

Given that the refinement of clinical thinking in healthcare relies on this deliberate practice^(7)^ and that digital information and communication technologies (DICT)^(9)^ could contribute to this issue, we have been working on developing a web platform aimed at providing users with the opportunity to practice clinical thinking in the care of patients with behavioral dysphonia, a specific communication difficulty that prevents the natural production of the voice and whose etiological factor is the inappropriate use of the voice or exposure to risk factors for vocal disorders^(10)^. The tool is called Script Voice, whose design encompasses the stages of assessment, diagnosis, and therapeutic planning, based on real clinical cases and arguments supported by the literature and the clinical thinking of experienced professionals. This paper aims to present the results of the initial stages of building Script Voz, which covered the planning, development of the platform, and preparation of content.

## METHOD

This is a methodological study aimed at developing a web platform that could guide users through simulated clinical care in speech therapy, using real clinical cases with a diagnosis of behavioral dysphonia. The research project was approved by the Research Ethics Committee of the Federal University of Minas Gerais under opinion 5.877.764. All the professionals involved in the study signed a Free and Informed Consent Form (FICF), as did the patients, who, by signing this document, authorized the use of their image, voice, and other personal data that will be available on the platform, which is open to access. The tool can be described as a pedagogical strategy using DICTs^(9)^. Following Falkembach's proposal^(11)^, the project will cover the following stages: planning the tool, developing the web platform, preparing the content, assessing the usability and user acceptance, redefining the tool, and defining the charges (preparing the user manual). This article provides descriptions of the first three stages, which have already been completed.

### Tool planning and web platform development

The planning of the platform began with two virtual meetings between two speech therapist researchers and a computer science professional, all higher education professors with more than 20 years of experience. Two computer engineering students also took part. The meetings were guided by the design thinking methodology^(12)^: the computer science professionals addressed questions to the researchers, seeking to understand the objectives of the intended tool, as well as the required functionalities.

### Content development

In addition to the aforementioned researchers, the content development stage of the platform also included two other speech therapists specializing in voice, who also have over 20 years of clinical and teaching experience. The four speech therapists concerned were from three different Brazilian states: two from Minas Gerais, one from Paraíba, and one from São Paulo. Five virtual meetings were held, each lasting an average of two hours, during which consensus was reached on three of the contents of the tool: assessment, diagnosis, and therapeutic planning scripts for the patients who will be part of the tool.

The discussions of the group of experts started with initial proposals, presented in PowerPoint, guided by one of the researchers, and shared with the other professionals. “Consensus” was defined as the agreement of at least three experts regarding the exclusion, adjustment, or inclusion of some component of each of the three aforementioned scripts.

## RESULTS

### Tool planning and web platform development

After gathering the requirements for developing the platform, the Computer Engineering professionals created prototypes of the web platform, initially low fidelity (basic design proposal) and later high fidelity (interactive representation of the platform, with greater similarity to the final design), using the Figma tool. These prototypes were developed within the NextJS framework, using the Strapi content management system. The videos were processed in Filmora editing software. The prototypes were refined interactively through usability tests of the platform, carried out by the researchers, allowing for adjustments and improvements based on the feedback received. Once approved, the high-fidelity prototype of Script Voz was hosted on the official website of the educational institution hosting the research.

In terms of functionality, after registering on the web platform, the user will be able to select one of the available clinical cases at a time and will then be led sequentially through the stages of assessment, diagnosis, and therapeutic planning of the case. Once the patient has been selected, the user must choose the answer(s) they consider appropriate to each of the questions proposed in each stage, amounting to a total of ten to fulfill the clinical thinking of each clinical case.

Therefore, the web platform will allow users to make clinical decisions based on guided reflection^(13)^, based on positive or negative feedback, and whether the answer(s) chosen for each of the questions is right or wrong. The feedback – to be provided in writing – will be supported by specialized literature and the clinical thinking of the specialists responsible for preparing the content of the simulator.

Script Voz will be aimed at speech therapy students and professionals interested in developing clinical thinking in the care of patients with behavioral dysphonia.

### Content development

Initially, the web platform will include six clinical cases of behavioral dysphonia, covering the otorhinolaryngological diagnoses of vocal polyp, sulcus, cyst, nodules, Reinke's edema, and posterior middle triangular cleft. Behavioral dysphonias were selected due to their higher prevalence in the general population compared to organic dysphonias^(14)^. Chart 1 shows the scripts for otorhinolaryngological assessment and speech therapy anamnesis for the clinical cases that will comprise the Script Voz web platform. Chart 2 shows the scripts for perceptual and acoustic assessments, as well as for vocal self-assessment. Chart 3 shows the scripts for establishing a diagnosis and therapeutic planning for clinical cases. These contents reflect the consensus of the four voice specialists who participated in the study.

**Chart 1:** Scripts for otorhinolaryngological assessment and speech therapy anamnesis for clinical cases on the Script-Voz web platform

**ENT assessment (videolaryngostroboscopy)**	
**Parameters**	**Classification**
**1. Vibrating pattern of the vocal folds** ^(15)^	
**a. Glottal closure**	Glottal closure: complete, cleft (anterior fusiform, central posterior, posterior triangular, middle posterior, hourglass, double, irregular, or parallel).
**b. Free edge contour of vocal folds**	Normal, convex, concave, irregular (right-left)
**c. Mucus movement**	Present, absent, decreased, increased, symmetrical, or asymmetrical.
**d. Supraglottic activity**	Anteroposterior, medial-lateral constriction (present - absent - unilateral - right - left, bilateral).
**2. Laryngeal injury** ^(16)^	Glottal closure: complete, cleft (anterior fusiform, central posterior, posterior triangular, middle posterior, hourglass, double, irregular, or parallel).
**Speech and hearing assessment** ^(10,17)^	
**Anamnesis items**	**Subitems**
**1. Personal information**	Name initials, age, date of birth, sex (male - female - non-binary), place of birth, schooling, profession, period of employment, if a teacher, please indicate cycle of education, length of employment.
**2. Past History of Dysphonia**	Onset (abrupt - progressive - could not provide information, evolution time (literal description), vocal stability (present-absent). If absent, worsening period, complaint (literal description), current vocal limitations.
**3. Complaint**	Literal description.
**4. Vocal behavior**	Vocal demand type: professional (spoken-sung) – social (spoken-sung), time and environment of vocal use.
**5. Vocal symptoms**	Voice Disorder Screening Index (VDSI)^(18)^
**6. Other symptoms**	Factors that interfere with vocal quality (improve or worsen the voice) – description.
**7. Habits – Risk factors for dysphonia**	Smoker (yes - no - time), former smoker (yes - no - time), drug user (yes - no - frequency - type), former drug user (yes - no - time), shouts frequently (yes - no), talks for a long time (yes - no - place), talks loudly (yes - no - frequency - place, intensity), has a busy social life (yes - no), works in a noisy environment (yes - no - which), works in a polluted environment (yes - no - which), is sensitive to changes in temperature (yes - no - how), self-medicates for voice problems (yes - no - how), drinks water regularly (yes - no - quantity), uses the voice in inappropriate body postures (yes - no - how).
**8. Complementary investigation**	General health: mental health, hearing health, allergies, physical activity (type - frequency), diet, GERD, upper airway infections, hormones, lung disorders, medication, family history, previous voice treatments.

**Chart 2:** Scripts for perceptual-auditory, acoustic, and vocal self-assessment of clinical cases for the Script-Voz web platform

**Vocal Perceptual-Hearing Assessment** ^(10,17)^		
**Sample type**	**Sample characterization**	**Collection details**
**1. Audio**	Sustained vowels at maximum phonation times.	Vowels /a/ and /ε/ three times each – maximum average times of phonation.
	Counting of numbers.	1 to 20
	Months of the year.	January to December
	Dynamic field.	Vowel /a/ habitual-strong-weak: auditory perceptual assessment and objective measurement by a decibel meter, starting with the weakest, except whisper, and the strongest, except shout; habitual-bass-sharp: the lowest sustained (thick), the highest (thin) sustained in isolation and glissando^(15,19)^.
**2. Audio and video**	Semi-spontaneous speech (trunk and face framing).	Answer to the question: *“What do you think of your voice?”*
**Vocal Acoustic Assessment** (VOXmetria/ VOXplot)^(10,17)^		
**Vocal sample**	**Analysis modules**	**Parameters**
**1. Sustained vowel /ε/ou /a/** (depending on the software)	Vocal analysis data.	Fundamental frequency (F0), jitter, shimmer, noise, irregularity, GNE ratio, NHR (average).
	Acoustic spectrography (narrow band).	Harmonics (track shape, degree of darkening, stability, presence of noise, presence of harmonics, sub-harmonics)^(16,20)^.
	Cepstral analysis.	CPPS, CPP.
**Chained speech**	Speaking voice.	Average fundamental frequency, average intensity.
**Laryngeal palpation** ^(21)^		
**Parameter**	**Structure – Region**	**Classification**
**Strength**	Right - left sternocleidomastoid muscles, supralaryngeal region, laryngeal resistance to lateral pressure.	Between 1 and 5: 1 is the minimum and 5 is the maximum.
**Position**	Larynx.	Sustained high (1), neutral (2), lowered (3), forced down (4).
**Vocal self-assessment**	**Focus**	
**Vocal Symptoms Scale (VSS)** ^(22)^	Symptomatology	
**Vocal Disadvantage Index (VDI)** ^(23)^	Quality of life	
**Dysphonia Coping Strategies Protocol (PEED)** ^(24)^	Cognition	

**Chart 3:** Scripts for establishing diagnosis and therapeutic planning for clinical cases on the Script Voz web platform

**Parameter**	**Diagnosis**	**Therapeutic planning**	
**Perceptual-auditory**	**Manifestations**	**Specific goals (Targets)**	**Strategies (Ingredients)** ^(25-27)^
**Vocal Quality (GRBASI)**	Hoarse, rough, breathy, asthenic, tense, unstable voice in mild, moderate, or intense degrees.	Adapting vocal quality to meet social and/or professional demands	General objective of therapeutic planning
**Average Maximum Phonation Times (MPT)**	Reduced or increased MPT.	Adjusting maximum phonation times	Techniques: vowel emission, fricative sound, lip or tongue vibration, nasal sounds – with maximum comfortable support.
**Dynamic Field**	1. Reduced dynamic range for frequency variation.	Adjusting dynamic range for frequency variation	Techniques: frequency modulation with variation towards the bass, frequency modulation with variation towards the treble, and musical scales (ascending and descending) with facilitating sounds.
	2. Reduced dynamic range for intensity variation.	Setting the dynamic range for intensity variation	Intensity modulation technique with facilitating sounds
**Resonance**	1. Low vocal resonance.	Balancing resonance	1. Techniques: nasal sounds, yawning-sighing, chewing associated with nasal sounds, monitoring by multiple routes - proprioceptive route.
	2. High vocal resonance.	Balancing resonance	Techniques: over-articulation, prolonged “b”, basal sound nasal sounds.
**Vocal attacks**	1. Sudden vocal attacks.	Matching vocal attacks	1. Techniques: control of sudden vocal attacks (emission of vowels initiated by aspirated vocal attacks), fricative sounds (alternating from unvoiced to voiced).
	2. Aspirated vocal attacks.	Matching vocal attacks	Techniques: control of aspirated vocal attacks (emission of vowels with a glottal blow), plosive sounds (pa ta ka), trigger sounds, sounded incomplete swallowing.
**Pitch**	1. Low pitch for gender and age.	Matching pitch to age and gender	1. Techniques: digital manipulation of the larynx, frequency modulation (towards the high end), hyperacute sound, musical scales (ascending), monitoring by multiple routes (auditory and visual).
	2. High pitch for gender and age.	Matching pitch to age and gender	Techniques: digital manipulation of the larynx, prolonged “b”, frequency modulation (towards the bass), musical scales (descending), basal sound.
**Loudness**	1. High loudness for the communicative context.	Adjusting loudness to the communicative context	Techniques: auditory repetition – Loop, sound amplification, intensity modulation, multi-way monitoring (auditory and visual).
	2. Reduced loudness for the communicative context.	Adjusting loudness to the communicative context	Techniques: auditory repetition – Loop, sound amplification, intensity modulation, multi-path monitoring (auditory and visual).
**Speech sound articulation**	1. Undifferentiated	Adjusting the articulation of speech sounds	Techniques: over-articulation, vowel-only reading, mouth opening, chewed speech, multi-path monitoring (visual and proprioceptive).
	2. Locked	Matching articulatory accuracy	
	3. Exaggerated	Matching articulatory accuracy	Multi-way monitoring technique – visual and proprioceptive.
**Speech rate**	1. Reduced speech rate.	Matching speech rate to the communicative context	Techniques: auditory repetition – Loop, vocal pacing, multi-pathway monitoring (auditory, visual, and proprioceptive).
	2. Increased speech rate.	Matching speech rate to the communicative context	Techniques: delayed auditory monitoring, auditory repetition – Loop, vocal pacing, multi-path monitoring (auditory, visual, and proprioceptive).
**Speech intelligibility**	Inadequate speech intelligibility.	Promoting speech intelligibility	Techniques: reading vowels only, over-articulation, delayed auditory monitoring, chewed speech, multiple pathway monitoring (visual and proprioceptive), tongue twisters.
**Speech rhythm**	Inappropriate speech rhythm.	Matching speech rhythm to the communicative context	Techniques: auditory repetition, vocal pacing, delayed auditory monitoring, multi-pathway monitoring (auditory and proprioceptive).
**Respiratory support during speech**	1. Upper respiratory type (clavicular).	Adjusting the type of respiratory support	Multi-way monitoring technique (visual and proprioceptive), breathing exercises: sustained inhalation, fractionated inhalation, fractionated inhalation + upper limbs. Breathing training for flow and force with respiratory stimulators/exercisers. Fricative sounds technique at maximum phonation time.
	2. Medium respiratory type (thoracic).	Adjusting the type of respiratory support	
**Breathing mode while speaking or singing**	1. Nasal breathing mode.	Setting up oro-nasal breathing	Breathing exercises with air injections through the oral cavity associated with nostril occlusion.
	2. Oral breathing mode.	Setting up oro-nasal breathing	Multi-way monitoring technique – Proprioceptive. Nasal aeration exercise with one nostril occluded (alternating nostrils).
**Respiratory coordination and articulation of speech sounds**	Respiratory and articulatory incoordination of speech sounds	Promoting respiratory and articulatory coordination of speech sounds	Techniques: proprioceptive monitoring, vibration of the lips or tongue in TMF, fricative sounds in TMF, nasal sounds in TMF, multi-way monitoring (proprioceptive and visual), modulated voice.
**Parameter**	**Diagnosis**	**Therapeutic planning**	
**Laryngeal palpation** Brito et al.^(21)^	**Manifestations**	**Specific goals (Targets)**	**Strategies (Ingredients)** ^(17)^
**Strength**		Adjusting muscle resistance.	1. Technique of cervical movements, stretching of the cervical muscles, massage of the cervical region (manual – electric massager), application of heat, technique of digital manipulation of the larynx.
**Laryngeal position**		Adjusting laryngeal position.	Techniques: digital manipulation of the larynx, cervical movements, stretching of the cervical muscles, massage of the cervical region (manual – electric massager), application of heat.


**Parameter**	**Diagnosis**	**Therapeutic planning**	
**ENT diagnosis**	**Etiological diagnosis**	**Specific goals (Targets)**	**Strategies (Ingredients)** ^(17)^
**1. Nodules 2. Polyp 3. Reinke’s edema 4. Cyst 5. Furrow**		1 to 6. Stimulating the mucus movement.	1 to 6. Techniques: vibrating sounds, basal sound, massager associated with glottal sonorization, fricative sounds (in sonority passage), phonation in tubes submerged in water.
		1, 2, and 5. Absorbing the injury.	1, 2, and 5. Techniques: lip and tongue vibration, voiced fricative sounds. Techniques: lip constriction, phonation in tubes.
		1 and 6. Adjusting glottal absorption.	1 and 6. Techniques: tongue or lip vibration, fricative sounds (in sonority passage). Techniques: inspiratory phonation, prolonged “b”, phonation in tubes.
**Supraglottic activity**		Removing the involvement of supra-glottic structures in phonation.	Techniques: inspiratory “i”, yawn-sigh, sniff, breath, high-pitched sound. Lip constriction sequence.
**Parameter**	**Diagnosis**	**Therapeutic planning**	
**Vocal self-assessment Protocols**	**Diagnosis**	**Specific goals (Targets)**	**Strategies (Ingredients)** ^(25,28)^
**Vocal Symptoms Scale (VSS)** ^(22)^	Above 16 points – self-perception of negative impact on voice functionality.	Removing and/or reducing vocal symptoms.	Pedagogy and Counseling. Vocal guidance – customized indirect vocal therapy based on approaches to deal with the patient’s symptoms.
**Vocal Disadvantage Index (VDI)** ^(23)^	Above 19 points – self-perception of vocal disadvantage.	Improving the voice-related patient’s quality of life.	Pedagogy and Counseling. Voice guidance – customized indirect voice therapy.
**Dysphonia Coping Strategies Protocol (PEED)** ^(24)^	Average score for individuals with voice complaints: 51.86. Average score for the population without vocal complaints: 23.18.	Promoting coping strategies for the solution of dysphonia.	Pedagogy and Counseling. Voice guidance – customized indirect voice therapy.

## DISCUSSION

Clinical simulation guides are didactic tools based on an academic consensus among professors, aimed at standardizing criteria to create clear concepts and contribute to educational projects that can address the curriculum’s need for problem-solving^(29)^. Simulation guides are divided into procedural and study guides. The former indicates the steps to be followed in clinical procedures. Study guides, on the other hand, consist of a structured instrument aimed at applying the student's previous knowledge to specific clinical situations, supported by the clinical problem and the simulator^(29)^.

In this proposal, the three step-by-step scripts for completing the assessment, diagnosis, and therapeutic planning stages of the clinical cases comprise the procedural guides, which have already been completed and presented in this article. The study guides, meanwhile, consist of real clinical cases with their respective questions and feedback, duly agreed by the panel of experts, as well as the tool itself, developed using digital information and communication technology (DICT).

The clinical thinking process in the speech therapy assessment of dysphonia requires gathering the patient's history and information about their vocal behavior, the subject's self-assessment of the impact of their problem in their personal and professional contexts, the perceptual and acoustic vocal analyses, the physical examination, and the correlation of all this information with the clinical examination and the otorhinolaryngological diagnosis^(10)^. This understanding guided the definitions of the data collection proposals that comprise the assessment script designed in this study by the specialists.

This complex process of analysis during the assessment often results in the identification of multiple signs and symptoms of the patient, which form the so-called clinical, manifestation, or syndromic diagnosis, which in turn differs from the etiological diagnosis, describing the cause of the illness^(30)^.

Once the diagnosis has been established, the signs and symptoms detected are addressed therapeutically in an integrated manner, aiming to improve the patient's vocal quality^(26)^. The specialists proposed guiding the user’s clinical thinking in establishing the diagnosis of manifestation based on a multidimensional assessment of each patient's voice, presenting the signs and symptoms systematically, before the therapeutic planning stage.

Vocal rehabilitation, in turn, is based on customized therapeutic planning and is described as a non-linear process of changes in behavior, muscle adjustments, or vocal identity^(10,29)^. The major challenge for speech therapists is to conduct effective treatment using the best scientific evidence available^(10)^. In addition to the intervention results being influenced by psychosocial, behavioral, environmental, and self-perception factors, voice specialists deal with a wide variety of vocal and laryngeal manifestations, which makes clinical thinking and therapeutic choices even more complex^(28)^. Moreover, speech therapists need to be aware of the effect (or expected effect) of their actions on the vocal physiology and communication of their patients^(31)^. These premises provided the guiding thread for developing the content of the therapeutic planning stage of the web platform: the specialists used the traditional basis of clinical thinking in their approach to dysphonia, with customized direct and indirect therapies^(10)^, adding knowledge of the Rehabilitation Treatment Specification System (SETR-Voz)^(27)^.

A clear understanding of the mental process followed by experienced professionals to make decisions in their professional practice is essential to guide the training of students, as well as to recognize their difficulties in clinical reasoning^(4)^. The proposed tool seeks to meet both objectives by allowing the user to be guided by the clinical thinking of experienced professionals. For each choice of answer to the proposed questions made by the user, the system will trigger positive or negative feedback, based on scientific literature, encouraging them to reinforce a certain piece of knowledge or to reflect and rethink their choices.

Despite the large number of studies covering clinical thinking in healthcare, few focus specifically on speech therapy. The literature points to efforts in Australia^(32-34)^, Chile^(35,36)^, the United States^(37)^, South Africa^(38)^, Ireland^(39)^, Spain^(40)^, and the United Kingdom^(4)^. These studies point to the importance of learner autonomy in the learning process and the need for proposals to train students based on case studies, enabling the development of clinical reasoning skills that are close to the mechanisms used by specialists^(32,35)^. Practitioners’ critical thinking and use of clinical instincts are given priority, suggesting a preponderance of these over the assessment measures themselves^(38)^.

It is worth highlighting that speech therapy practice is patient- and professional-centered and that critical thinking is a component of decision-making, providing a tool to address the different ingredients and the dynamic nature of clinical practice^(39)^. The newly designed web platform seeks to meet these recommendations by 1) shedding light on experienced professionals' clinical thinking; 2) using real clinical cases; 3) fostering critical thinking and reflection in learning; and 4) encouraging science-based decision-making: 1) sheds light on the clinical reasoning of experienced professionals; 2) uses real clinical cases; 3) values critical thinking and reflection in learning and; 4) encourages science-based decision making.

The clinical scenarios – in the final stages of development – will guide the user's experience with the web platform through questions and feedback based on their own decision-making, thus completing the study guide for the Script Voz platform. Next, the tool will be subject to usability and user acceptance evaluations, redefinition, and pricing, at which point it can be released to the public. Initially fed with six clinical cases, it is expected to be updated gradually with new clinical scenarios, becoming an increasingly diverse teaching strategy in the field of speech. The web platform is also expected to be translated into English, thereby broadening its reach.

## CONCLUSION

The platform was developed using the Figma tool, based on the Design Thinking methodology^(12)^. Script Voz will be open access and its high-fidelity prototype is already hosted on the website of the educational institution hosting the research (UFMG). The platform’s procedural guides were drafted based on the consensus of a panel of experts. These guidelines are designed to guide the procedures for collecting data, establishing diagnoses of manifestations, therapeutic planning, and speech therapy approaches for the clinical cases that will feature in the tool. The questions and feedback that will guide the user experience on the web platform are currently in the final stages of development. The tool will use real clinical cases and arguments supported by the literature and the clinical thinking of experienced professionals.
