# Supplementary material for: Script Voz – clinical case simulator of adults with behavioral dysphonia: planning and content creation
Source: Codas. 2025 Feb 3;37(1):e20240075. doi: 10.1590/2317-1782/e20240075en (PMC11801194; doi:10.1590/2317-1782/e20240075en)
Supplement: Translation

# Translation: *Script Voz* – simulador de casos clínicos de adultos com disfonia comportamental: planejamento e elaboração de conteúdo

**DOI:** 10.1590/2317-1782/e20240075pt

**Authors:** Lorena Luiza Costa Rosa Nogueira, Sandro Renato Dias, Anna Alice de Figueirêdo Almeida, Renata Rangel Azevedo, Ana Cristina Côrtes Gama

**Affiliations:** 1 Departamento de Fonoaudiologia, Universidade Federal de Minas Gerais – UFMG - Belo Horizonte (MG), Brasil.; 2 Departamento de Computação, Centro Federal de Educação Tecnológica de Minas Gerais – CEFET-MG – Belo Horizonte (MG), Brasil.; 3 Departamento de Fonoaudiologia, Universidade Federal da Paraíba – UFPB- João Pessoa (PB), Brasil.; 4 Departamento de Fonoaudiologia, Universidade Federal de São Paulo – UNIFESP - São Paulo (SP), Brasil.

**Keywords:** Fonoaudiologia, Raciocínio Clínico, Tomada de Decisão Clínica, Cognição, Aprendizagem, Treinamento por Simulação

## Abstract

**Objetivo:**

Apresentar os resultados preliminares do desenvolvimento de uma plataforma web que visa o treino do raciocínio clínico voltado para a abordagem fonoaudiológica de pacientes com disfonia comportamental.

**Método:**

Estudo metodológico que descreve as etapas de desenvolvimento da ferramenta. Os conteúdos foram definidos por consenso a partir de painel de especialistas. O projeto cumprirá com as etapas de planejamento, desenvolvimento da plataforma, elaboração do conteúdo, avaliação de usabilidade e aceitação dos usuários, redefinição da ferramenta e determinação dos encargos. O presente artigo apresenta os processos e os resultados das três primeiras etapas.

**Resultados:**

Denominada Script Voz, a plataforma web contemplará, inicialmente, seis casos clínicos de disfonias comportamentais. O usuário poderá selecionar um caso clínico de cada vez, devendo ser conduzido sequencialmente pelas etapas de avaliação, diagnóstico e planejamento terapêutico do caso. Para tanto, deverá escolher a(s) resposta(s) que julgar adequada(s) a cada uma das dez perguntas propostas para cada caso clínico. São apresentados os guias de procedimentos para avaliação, diagnóstico e planejamento terapêutico voltados para os casos clínicos que comporão a ferramenta, evidenciando os seus processos.

**Conclusão:**

A Script Voz será de livre acesso. Os guias de procedimento aqui dispostos nortearão a coleta de dados, os diagnósticos de manifestação e o planejamento terapêutico voltados para os casos clínicos que comporão a ferramenta. As perguntas e devolutivas que guiarão a experiência do usuário encontram-se ainda em fase final de elaboração. A ferramenta usará casos clínicos reais e argumentos ancorados no raciocínio clínico de profissionais experientes.

## INTRODUÇÃO

O raciocínio clínico em saúde é o processo cognitivo que possibilita ao profissional estabelecer diagnóstico correto e conduta adequada diante de um determinado problema clínico^(1)^. As dificuldades de raciocínio clínico têm sido relatadas em muitas profissões, especialmente na medicina. Estudo revela que os obstáculos enfrentados por profissionais inexperientes e estudantes se relacionam tanto ao conhecimento propriamente dito, quanto à estratégia em tarefas que envolvem esse raciocínio^(2)^.

O Conselho Nacional de Saúde estabelece que a formação do fonoaudiólogo deve dotar o profissional dos conhecimentos requeridos para o exercício da promoção, prevenção e recuperação em todos os níveis de atenção^(3)^. Entre as competências mencionadas, ressaltam-se: fazer avaliações, formular diagnósticos diferenciais e realizar manejo terapêutico, buscando promover cuidado centrado nas necessidades dos usuários, da família e da comunidade, em todos os ciclos de vida^(3)^.

Pesquisas apontam dificuldades de raciocínio clínico de graduandos em Fonoaudiologia ao fazerem diagnósticos^(4,5)^. As diferenças relacionadas a esta habilidade também se mostraram importantes quando comparados estudantes com profissionais fonoaudiólogos; a desigualdade diz respeito ao melhor planejamento de hierarquia de avaliações e a melhores conexões entre avaliação e planejamento terapêutico pelos mais experientes^(5)^.

A literatura aponta as teorias que buscaram esclarecer o sistema de construção do raciocínio clínico ao longo do tempo, partindo da teoria processual. De acordo com ela^(1)^, diante de um caso clínico, os profissionais de saúde estabelecem hipóteses diagnósticas, as quais são acatadas ou refutadas a partir de novas informações. Tal teoria, no entanto, não explicou as diferenças de competência entre profissionais experientes e novatos, dando lugar à teoria estrutural^(6)^. Esta, por sua vez, postula que o raciocínio clínico é dependente do conhecimento adquirido, no entanto, não basta acumular conhecimento, é preciso que ele seja estruturado na memória de modo a favorecer o seu uso no dia a dia, premissa que abriu espaço para a proposição de dois tipos de raciocínio: o analítico e o não-analítico^(7)^. O primeiro é utilizado na solução de problemas mais complexos, quando entra em ação o método hipotético-dedutivo; já o raciocínio não-analítico é empregado na resolução de casos clínicos mais cotidianos, aos quais o profissional já tenha sido exposto repetidas vezes, tendo criado o que os autores chamaram de “*scripts* de doenças”^(1)^. A expertise, então, vai se consolidando a partir da prática intensa de atendimentos. A teoria da construção dos *scripts* de doenças parte do pressuposto de que, para construir o raciocínio clínico, é preciso articular o conhecimento de disciplinas básicas às apresentações clínicas das doenças (sinais e sintomas) e à exposição a problemas clínicos reiteradamente^(7)^. De acordo com essa teoria, o aspecto mais crítico da aprendizagem é a prática deliberada, capaz de enriquecer o repertório de conceitos e aprimorar o depósito de problemas já resolvidos^(6)^.

A simulação clínica vem sendo utilizada na área da saúde como estratégia de aprendizagem significativa, permitindo ao aprendiz participar ativamente da construção do seu conhecimento e configurando alternativa para a ampliação do repertório de *scripts* de doenças^(8)^. É definida como uma técnica ou uma tecnologia que busca recriar as particularidades de situações de vida real, viabilizando a aquisição de habilidades e competências em ambiente seguro, antes do contato direto com os pacientes^(8)^.

Considerando que o amadurecimento do raciocínio clínico em saúde é dependente da mencionada prática deliberada^(7)^ e que as tecnologias digitais de informação e comunicação (TDIC)^(9)^ podem trazer contribuições à temática, os autores do presente artigo vêm se dedicando ao desenvolvimento de uma plataforma *web* que visa oportunizar ao usuário o treino do raciocínio clínico no atendimento fonoaudiológico a pacientes com disfonias comportamentais, dificuldade específica da comunicação, que impede a produção natural da voz e que tem como fator etiológico o uso inadequado da voz ou a exposição a fatores de risco para distúrbios vocais^(10)^. A ferramenta foi denominada *Script* Voz. Sua concepção permeia as etapas de avaliação, diagnóstico e planejamento terapêutico, utilizando-se de casos clínicos reais e argumentos ancorados na literatura e no raciocínio clínico de profissionais experientes. O presente artigo tem como objetivo apresentar os resultados das etapas iniciais de construção da *Script* Voz, as quais envolveram o planejamento, o desenvolvimento da plataforma e a elaboração de conteúdo.

## MÉTODO

Trata-se de estudo metodológico voltado para o desenvolvimento de uma plataforma *web* com potencial de guiar o usuário no atendimento clínico simulado em Fonoaudiologia, com utilização de casos clínicos reais com diagnóstico de disfonia comportamental. O projeto de pesquisa foi aprovado pelo Comitê de Ética em Pesquisa da Universidade Federal de Minas Gerais sob parecer 5.877.764. Todos os profissionais envolvidos no estudo assinaram Termo de Consentimento Livre e Esclarecido (TCLE), bem como os pacientes, que, ao assinarem o referido documento, autorizaram o uso de imagem, voz e demais dados pessoais que estarão disponíveis na plataforma, de livre acesso. A ferramenta pode ser traduzida como uma estratégia pedagógica com uso de TDIC^(9)^. Seguindo a proposta de Falkembach^(11)^, o projeto cumprirá com as seguintes etapas: planejamento da ferramenta, desenvolvimento da plataforma *web*, elaboração do conteúdo, avaliação de usabilidade e aceitação dos usuários, redefinição da ferramenta e determinação dos encargos (elaboração do manual do usuário). O presente artigo apresenta as descrições das três primeiras etapas, já concluídas.

### Planejamento da ferramenta e desenvolvimento da plataforma web

O planejamento da plataforma teve início com duas reuniões virtuais, envolvendo duas pesquisadoras fonoaudiólogas e um profissional da ciência da computação, todos professores do ensino superior com mais de 20 anos de experiência. Foram envolvidos também dois estudantes de Engenharia da Computação. Os encontros foram norteados pela metodologia *design thinking* ^(12)^: os profissionais da área da computação direcionaram perguntas às pesquisadoras, buscando compreender os objetivos da ferramenta pretendida, bem como as funcionalidades requeridas.

### Elaboração do conteúdo

A etapa de elaboração do conteúdo da plataforma envolveu, além das pesquisadoras anteriormente mencionadas, outras duas fonoaudiólogas especialistas em voz, também com mais de 20 anos de experiência clínica e docente. As quatro fonoaudiólogas envolvidas eram de três diferentes estados do Brasil: duas de Minas Gerais, uma da Paraíba e uma de São Paulo. Foram realizadas cinco reuniões virtuais, com duração média de duas horas cada, por meio das quais foram estabelecidos consensos relacionados a três conteúdos da ferramenta: roteiros de avaliação, de diagnóstico e de planejamento terapêutico voltados para os pacientes que comporão a ferramenta.

As discussões do grupo de especialistas tiveram como ponto de partida proposições iniciais, apresentadas em *power point,* norteadas por uma das pesquisadoras e compartilhadas com as demais profissionais. Definiu-se por “consenso” a concordância de, no mínimo, três especialistas em relação à exclusão, ao ajuste ou à inclusão de algum componente de cada um dos três roteiros mencionados.

## RESULTADOS

### Planejamento da ferramenta e desenvolvimento da plataforma web

Após coleta dos requisitos para elaboração da plataforma, os profissionais da Engenharia da Computação criaram protótipos da plataforma *web*, inicialmente de baixa fidelidade (proposição de desenho básico) e posteriormente de alta fidelidade (representação interativa da plataforma, com maior semelhança com o *design* final), utilizando a ferramenta *Figma*. Tais protótipos foram desenvolvidos com o *framework NextJS*, usando o sistema de gerenciamento de conteúdo *Strapi*. Os vídeos foram tratados usando o *software* de edição *Filmora*. Os protótipos foram refinados interativamente por meio de testes de usabilidade da plataforma, realizados pelas pesquisadoras, permitindo ajustes e melhorias a partir dos *feedbacks* recebidos. Após aprovação, a prototipagem de alta fidelidade da *Script Voz* foi hospedada no *site* oficial da instituição de ensino que sedia a pesquisa.

No que diz respeito à funcionalidade, após cadastro na plataforma *web*, o usuário poderá selecionar um dos casos clínicos disponíveis de cada vez, devendo ser, então, conduzido sequencialmente pelas etapas de avaliação, diagnóstico e planejamento terapêutico do caso. Uma vez selecionado o paciente, o usuário deverá escolher a(s) resposta(s) que julgar adequada(s) a cada uma das perguntas propostas em cada etapa, totalizando dez para cumprir com o raciocínio clínico de cada caso clínico.

A plataforma *web* possibilitará, portanto, tomadas de decisões clínicas por parte do usuário baseadas em reflexão guiada^(13)^, a partir de devolutivas positivas ou negativas, pelo acerto ou erro da(s) resposta(s) escolhida(s) para cada uma das perguntas. As devolutivas – a serem oferecidas por escrito - serão fundamentadas pela literatura especializada e ancoradas no raciocínio clínico das especialistas responsáveis pela elaboração dos conteúdos do simulador.

A *Script* Voz terá como público-alvo alunos de fonoaudiologia e profissionais interessados em desenvolver o raciocínio clínico voltado ao atendimento de pacientes com disfonia comportamental.

### Elaboração do conteúdo

A plataforma *web* contemplará, inicialmente, seis casos clínicos de disfonias comportamentais, envolvendo os diagnósticos otorrinolaringológicos de pólipo vocal, sulco, cisto, nódulos, edema de *Reinke* e fenda triangular médio posterior. A escolha pelas disfonias comportamentais deveu-se à maior prevalência dessas na população em geral, comparada às orgânicas^(14)^. O Quadro 1 apresenta os roteiros para avaliação otorrinolaringológica e anamnese fonoaudiológica voltados para os casos clínicos que comporão a plataforma *web* Script Voz. O Quadro 2 contempla os roteiros para realização das avaliações perceptivo-auditiva, acústica, bem como para a autoavaliação vocal. O Quadro 3 apresenta os roteiros para estabelecimento de diagnóstico e planejamento terapêutico direcionados aos casos clínicos. Tais conteúdos refletem o consenso entre as quatro especialistas em voz envolvidas no estudo.

**Quadro 1:** Roteiros para avaliação otorrinolaringológica e anamnese fonoaudiológica voltados para casos clínicos da plataforma *web Script*-Voz

**Avaliação otorrinolaringológica** (videolaringoestroboscopia)	
**Parâmetros**	**Classificação**
**1. Padrão vibratório de pregas vocais** ^(15)^	
**a. Fechamento glótico**	Completo, Fendas (fusiforme anterior, posterior central, triangular posterior, médio posterior, ampulheta, dupla, irregular, paralela)
**b. Contorno de borda livre de pregas vocais**	Normal, convexo, côncavo ou irregular (direita- esquerda)
**c. Movimento mucoondulatório**	Presente, ausente, diminuído, aumentado, simétrico, assimétrico
**d. Atividade supraglótica**	Constrição anteroposterior, médio-lateral (presente - ausente – unilateral - direita - esquerda, bilateral)
**2. Lesão laríngea** ^(16)^	Especificar tipo e localização
**Avaliação fonoaudiológica** ^(10,17)^	
**Itens anamnese**	**Subitens**
**1. Dados Pessoais**	Iniciais do nome, idade, data de nascimento, sexo (masculino – feminino - não binário), naturalidade, escolaridade, profissão, período de trabalho, se professor, especificar ciclo do ensino, tempo de profissão
**2. História Pregressa da Disfonia**	Forma de aparecimento (abrupta - progressiva - não soube informar, tempo de evolução (descrição literal), estabilidade vocal (presente-ausente). Se ausente, qual o período de piora, queixa (descrição literal), limitações vocais atuais
**3. Queixa**	Descrição literal
**4. Comportamentos Vocais**	Demanda vocal *tipo*: profissional (falada-cantada) – social (falada-cantada), tempo e ambiente de uso vocal
**5. Sintomas Vocais**	Índice de Triagem de Distúrbio da Voz (ITDV)^(18)^
**6. Outros Sintomas**	Fatores que interferem na qualidade vocal (melhoram ou pioram a voz) – descrição
**7. Hábitos – Fatores de risco para a disfonia**	Tabagista (sim – não – tempo), ex-tabagista (sim – não - tempo), usuário de drogas (sim – não – frequência - tipo), ex-usuário de drogas (sim – não – tempo), grita com frequência (sim – não), fala durante muito tempo (sim – não – local), fala em forte (sim – não - frequência – local, intensidade), tem vida social intensa (sim – não), trabalha em ambiente ruidoso (sim – não – qual), trabalha em ambiente poluído (sim – não – qual), é sensível a mudanças de temperatura (sim – não – como), se automedica para problemas da voz (sim – não – como), faz ingestão regular de água (sim – não - quantidade), usa a voz em posturas corporais inadequadas (sim – não – como)
**8. Investigação complementar**	Saúde geral: saúde mental, saúde auditiva, alergias, atividade física (tipo –frequência), alimentação, RGE, infecções de vias aéreas superiores, hormônios, distúrbios pulmonares, medicamentos, antecedentes familiares, tratamentos anteriores para voz

**Quadro 2:** Roteiros para avaliação perceptivo-auditiva, acústica e autoavaliação vocal de casos clínicos para plataforma *web Script*-Voz

**Avaliação Perceptivo- Auditiva Vocal** ^(10,17)^		
**Tipo de amostra**	**Caracterização da amostra**	**Detalhamento para coleta**
**1. Áudio**	Vogais sustentadas em tempos máximos de fonação	Vogais /a/ e /ε/ 3 tempos cada – média de tempos máximos de fonação
	Contagem de números	1 a 20
	Meses do ano	Janeiro a dezembro
	Campo dinâmico	Vogal /a/ habitual-forte-fraco: avaliação perceptivo auditiva e medida objetiva por decibelímetro, começando pelo mais fraco, excetuando sussurro e o mais forte, excetuando o grito; habitual-grave-agudo: o mais grave sustentado (grosso), o mais agudo (fino) sustentado isolados e glissando^(15,19)^.
**2. Áudio e vídeo**	Fala semi-espontânea (enquadramento tronco e rosto)	Resposta à pergunta: *“O que você acha da sua voz?”*
**Avaliação Acústica Vocal** (VOXmetria/ VOXplot)^(10,17)^		
**Amostra vocal**	**Módulos de análise**	**Parâmetros**
**1. Vogal /ε/ou /a/ sustentada** (a depender do *software*)	Dados da análise vocal	Frequência Fundamental (F0), *jitter, shimmer, r*uído, irregularidade, proporção GNE, NHR (média)
	Espectrografia acústica (faixa estreita)	Harmônicos (forma do traçado, grau de escurecimento, estabilidade, presença de ruído, presença de harmônicos, sub-harmônicos)^(16,20)^
	Análise Cepstral	CPPS, CPP
**Fala encadeada**	Voz na Fala	Frequência fundamental média, média de intensidade
**Avaliação Palpatória da Laringe ^(^** ^21)^		
**Parâmetro**	**Estrutura – Região**	**Classificação**
**Resistência**	Músculos esternocleidomastoideos direito - esquerdo, região supralaríngea, resistência laríngea à pressão lateral	Entre 1 e 5, sendo 1 mínima e 5 máxima
**Posição**	Laringe	Alta mantida (1), neutra (2), abaixada (3), abaixada forçada (4)
**Autoavaliação vocal**	**Enfoques**	
**Escala de Sintomas Vocais (ESV)** ^(22)^	Sintomatologia	
**Índice de Desvantagem Vocal (IDV)** ^(23)^	Qualidade de vida	
**Protocolo Estratégias Enfrentamento da Disfonia (PEED)** ^(24)^	Cognição	

**Quadro 3:** Roteiros para estabelecimento de diagnóstico e de planejamento terapêutico voltados aos casos clínicos da plataforma *web Script* Voz

**Parâmetro**	**Diagnóstico**	**Planejamento Terapêutico**	
**Perceptivo-auditivo**	**Manifestações**	**Objetivos específicos (Alvos)**	**Estratégias (Ingredientes)** ^(25-27)^
**Qualidade Vocal (GRBASI)**	Voz rouca, áspera, soprosa, astênica, tensa, instável em graus leve, moderado ou intenso	Adequar a qualidade vocal atendendo às demandas sociais e/ou profissionais	Objetivo geral do planejamento terapêutico
**Média Tempos Máximos de Fonação (TMF)**	TMF reduzidos ou aumentados	Adequar tempos máximos de fonação	Técnicas: emissão de vogal, som fricativo, vibração de lábios ou língua, sons nasais - em máxima sustentação confortável.
**Campo Dinâmico**	1. Campo dinâmico reduzido para variação de frequência	Ajustar campo dinâmico para variação de frequência	Técnicas: modulação de frequência com variação para o grave, modulação de frequência com variação para o agudo, de escalas musicais (ascendentes e descendentes) com sons facilitadores
	2. Campo dinâmico reduzido para variação de intensidade	Ajustar campo dinâmico para variação de intensidade	Técnica de modulação da intensidade com sons facilitadores
**Ressonância**	1. Ressonância vocal baixa	Equilibrar ressonância	1. Técnicas: som nasal, bocejo-suspiro, mastigatória associada a sons nasais, monitoramento por múltiplas vias - via proprioceptiva
	2. Ressonância vocal alta	Equilibrar ressonância	Técnicas: sobrearticulação, “b” prolongado, som basal sons nasais
**Ataques Vocais**	1. Ataques vocais bruscos	Adequar ataques vocais	1. Técnicas: controle de ataques vocais bruscos (emissão de vogais iniciadas por ataques vocais aspirados), sons fricativos alternância de não vozeado para vozeado)
	2. Ataques vocais aspirados	Adequar ataques vocais	Técnicas: controle de ataques vocais aspirados (emissão de vogais com golpe de glote), sons plosivos (pa ta ka), sons disparadores, deglutição incompleta sonorizada
***Pitch***	1. *Pitch* vocal grave para o gênero e a idade	Adequar o *pitch* à idade e ao gênero	1. Técnicas: manipulação digital da laringe, modulação de frequência (para o agudo), som hiperagudo, escalas musicais (ascendentes), monitoramento por múltiplas vias (auditiva e visual)
	2. *Pitch* vocal agudo para o gênero e a idade	Adequar o *pitch* à idade e ao gênero	Técnicas: manipulação digital da laringe, “b” prolongado, modulação de frequência (para o grave), escalas musicais (descendentes), som basal
***Loudness***	1. *Loudness* elevada para o contexto comunicativo	Adequar a *loudness* ao contexto comunicativo	Técnicas: repetição auditiva – *Loop,* amplificação sonora, modulação de intensidade, monitoramento por múltiplas vias (auditiva e visual)
	*2 Loudness* reduzida para o contexto comunicativo	Adequar a *loudness* ao contexto comunicativo	Técnicas: repetição auditiva-*Loop*, amplificação sonora, modulação de intensidade, monitoramento por múltiplas vias (auditiva e visual)
**Articulação dos sons da fala**	1. indiferenciada	Adequar a articulação dos sons da fala	Técnicas: sobrearticulação, leitura somente de vogais, abertura de boca, fala mastigada, monitoramento por múltiplas vias (visual e proprioceptiva)
	2. travada	Adequar precisão articulatória	
	3. exagerada	Adequar precisão articulatória	Técnica de monitoramento por múltiplas vias – visual e proprioceptiva
**Velocidade de Fala**	1. Velocidade de fala reduzida	Adequar a velocidade de fala ao contexto comunicativo	Técnicas: repetição auditiva -*Loop*, marca passo vocal, monitoramento por múltiplas vias (auditiva, visual e proprioceptiva)
	2. Velocidade de fala aumentada	Adequar a velocidade de fala ao contexto comunicativo	Técnicas: monitoramento auditivo retardado, repetição auditiva – *Loop*, marca passo vocal, monitoramento por múltiplas vias (auditiva, visual e proprioceptiva)
**Inteligibilidade de fala**	Inteligibilidade de fala inadequada	Favorecer a inteligibilidade de fala	Técnicas: leitura somente de vogais, sobrearticulação, monitoramento auditivo retardado, fala mastigada, monitoramento por múltiplas vias (visual e proprioceptiva), trava línguas
**Ritmo**	Ritmo de fala inadequado	Ajustar o ritmo de fala ao contexto comunicativo	Técnicas: repetição auditiva, marca passo vocal, monitoramento auditivo retardado, monitoramento por múltiplas vias (auditiva e proprioceptiva)
**Tipo respiratório durante a fala**	1. Tipo respiratório superior (clavicular)	Ajustar o tipo respiratório	Técnica de monitoramento por múltiplas vias (visual e proprioceptivo), exercícios respiratórios: inspiração sustentada inspiração fracionada, inspiração fracionada + membros superiores Treino respiratório para fluxo e força com incentivadores/ exercitadores respiratórios Técnica de sons fricativos em tempo máximo de fonação
	2. Tipo respiratório médio (torácico)	Ajustar o tipo respiratório	
**Modo respiratório durante a fala ou o canto**	1. Modo respiratório nasal	Instalar respiração oro-nasal	Exercício respiratório com injeção de ar pela cavidade oral associada a oclusão de narinas Técnica de monitoramento por múltiplas vias - proprioceptiva
	2. Modo respiratório oral	Instalar respiração oro-nasal	Exercício de aeração nasal com uma das narinas ocluídas (alternando narinas)
**CPFA durante a fala**	Incoordenação PFA	Favorecer a CPFA	Técnicas: monitoramento proprioceptivo, vibração de lábios ou língua em TMF, sons fricativos em TMF, sons nasais em TMF, monitoramento por múltiplas vias (proprioceptiva e visual), voz salmodiada
**Parâmetro**	**Diagnóstico**	**Planejamento Terapêutico**	
**Avaliação palpatória da laringe** Brito et al.^(21)^	**Manifestações**	**Objetivos específicos (Alvos)**	**Estratégias (Ingredientes)** ^(17)^
**Resistência**	Resistência aumentada de: músculos esternocleidomastoideo (D-E), e/ou região supralaríngea; e/ou laríngea à pressão lateral	Adequar resistência da musculatura	1. Técnica de movimentos cervicais, alongamentos musculatura cervical, massagem em região cervical (manual – massageador elétrico), aplicação de calor, técnica de manipulação digital da laringe
**Posição Laríngea**	1. Alta mantida	Ajustar posição laríngea	Técnicas: manipulação digital da laringe, movimentos cervicais, alongamentos musculatura cervical, massagem em região cervical (manual – massageador elétrico), aplicação de calor
	2. Abaixada		
	3. Abaixada forçada		
**Parâmetro**	**Diagnóstico**	**Planejamento Terapêutico**	
**Diagnóstico otorrinolaringológico**	**Diagnóstico Etiológico**	**Objetivos específicos (Alvos)**	**Estratégias (Ingredientes)** ^(17)^
**1.Nódulos 2. Pólipo 3. Edema de *Reinke* 4. Cisto 5. Sulco**	Disfonia comportamental	1 a 6. Estimular o movimento mucoondulatório	1 a 6. Técnicas: sons vibrantes, som basal, massageador associado à sonorização glótica, sons fricativos (em passagem de sonoridade), fonação em tubos submersos em água
		1, 2 e 5. Absorver lesão	1, 2 e 5. Técnicas: vibração de lábios e de língua, sons fricativos vozeados Técnicas: constrição labial, fonação em tubos
		1 e 6. Adequar a coaptação glótica	1 e 6. Técnicas: vibração de língua ou lábios, sons fricativos (em passagem de sonoridade) Técnicas: fonação inspiratória, “b” prolongado, fonação em tubos
**Atividade supraglótica**	Participação de estruturas supraglóticas à fonação	Eliminar participação de estruturas supra glóticas à fonação.	Técnicas: “i” inspiratório, bocejo-suspiro, *sniff*, sopro e som agudo Sequência de constrição labial
**Parâmetro**	**Diagnóstico**	**Planejamento Terapêutico**	
**Autoavaliação vocal Protocolos**	**Diagnóstico**	**Objetivos específicos (Alvos)**	**Estratégias (Ingredientes)** ^(25,28)^
**Escala de Sintomas Vocais (ESV)** ^(22)^	Acima de 16 pontos – autopercepção de impacto negativo na funcionalidade da voz.	Eliminar e/ou reduzir sintomas vocais	Pedagogia e Aconselhamento Orientação vocal– terapia vocal indireta customizada com abordagens para lidar com a sintomatologia do paciente.
**Índice de Desvantagem Vocal (IDV)** ^(23)^	Acima de 19 pontos - autopercepção de desvantagem vocal	Melhorar a qualidade de vida do paciente relacionada à voz	Pedagogia e Aconselhamento Orientação vocal – terapia vocal indireta customizada
**Protocolo Estratégias de Enfrentamento da Disfonia (PEED)** ^(24)^	Escore médio de indivíduos com queixa vocal: 51,86 Escore médio da população sem queixa vocal: 23,18	Favorecer a adoção de estratégias de enfrentamento adequadas à solução da disfonia.	Pedagogia e Aconselhamento Orientação vocal – terapia vocal indireta customizada

## DISCUSSÃO

Os guias de simulação clínica são ferramentas didáticas estabelecidas a partir de consenso acadêmico de professores, buscando unificar critérios para gerar conceitos claros e contribuir com projetos educacionais que possam responder às necessidades curriculares de resolução de problemas^(29)^. Entre os guias de simulação, distinguem-se os de *procedimento* e os de *estudo*. Os primeiros indicam o passo a passo a ser seguido nos procedimentos clínicos. Os guias de estudo, por sua vez, consistem no instrumento estruturado, que tem como objetivo a aplicação dos conhecimentos prévios do aluno a situações clínicas específicas, recorrendo a dois elementos de auxílio: o problema clínico e o simulador^(29)^. Na presente proposta, os três roteiros com o passo a passo para cumprimento das etapas de avaliação, diagnóstico e planejamento terapêutico dos casos clínicos constituem os guias de procedimento, já finalizados e apresentados no presente artigo. Os guias de estudo, por outro lado, consistem nos casos clínicos reais com as suas respectivas perguntas e devolutivas, devidamente consensuadas pelo painel de especialistas, além da própria ferramenta, elaborada a partir da tecnologia digital de informação e comunicação (TDIC).

O processo de raciocínio clínico na avaliação fonoaudiológica das disfonias envolve a obtenção do histórico do paciente a as informações sobre o seu comportamento vocal, a autoavaliação do sujeito sobre o impacto do seu problema nos contextos pessoal e profissional, as análises perceptivo-auditiva e acústica vocais, o exame físico e, finalmente, a correlação entre todas essas informações com o exame clínico e o diagnóstico otorrinolaringológico^(10)^. Tal entendimento guiou as definições das propostas de coleta de dados que compõem o roteiro de avaliação concebido no presente estudo pelas especialistas.

Este intrincado processo de análise, realizado durante a avaliação, resulta frequentemente na identificação de múltiplos sinais e sintomas do paciente, os quais compõem o chamado diagnóstico clínico, de manifestação ou sindrômico, e, por sua vez, diferencia-se do diagnóstico etiológico, que descreve a causa^(30)^.

Estabelecidos os diagnósticos, os sinais e sintomas identificados são abordados terapeuticamente de maneira entrelaçada, visando à melhora da qualidade vocal do paciente^(26)^. As especialistas envolvidas propõem a condução do raciocínio clínico do usuário no estabelecimento do diagnóstico de manifestação a partir da avaliação multidimensional da voz de cada paciente, apresentando os sinais e sintomas de forma sistematizada, previamente à etapa de planejamento terapêutico.

A reabilitação vocal, por sua vez, é conduzida a partir do planejamento terapêutico personalizado, sendo descrita como um processo não linear de mudanças de comportamento, de ajustes musculares ou de identidade vocal^(10,29)^. O maior desafio dos fonoaudiólogos é conduzir um tratamento efetivo, usando as melhores evidências científicas disponíveis^(10)^. Além dos resultados da intervenção poderem sofrer influências de fatores psicossociais, comportamentais, ambientais e auto perceptivos dos sujeitos, o especialista em voz lida com uma grande variedade de manifestações vocais e laríngeas, o que torna o raciocínio clínico e as escolhas terapêuticas ainda mais complexos^(28)^. É necessário, ainda, que o fonoaudiólogo, em seu processo de formação, passe a conhecer o efeito (ou as expectativas de efeito) de suas ações sobre a fisiologia vocal e a comunicação do seu paciente^(31)^. Tais premissas constituíram o fio condutor na elaboração dos conteúdos da etapa de planejamento terapêutico da plataforma *web*: as especialistas utilizaram a base tradicional do raciocínio clínico na abordagem das disfonias, com as terapias direta e indireta customizadas^(10)^, adicionando conhecimento do Sistema de Especificação do Tratamento de Reabilitação (SETR-Voz)^(27)^.

Entender de maneira clara o processo mental utilizado por profissionais experientes para a sua tomada de decisão no exercício profissional é fundamental para nortear a formação dos estudantes, tanto quanto identificar as suas dificuldades de raciocínio clínico^(4)^. A ferramenta proposta busca cumprir com os dois alvos, ao permitir que o usuário seja guiado pelo raciocínio clínico de profissionais experientes - a cada escolha de resposta às perguntas propostas feita pelo usuário, o sistema disparará uma devolutiva positiva ou negativa, ancorada na literatura científica, convidando-o a reforçar um determinado conhecimento ou a refletir e refazer as suas escolhas.

Apesar do grande número de estudos envolvendo o raciocínio clínico na área da saúde, ainda são poucos os dedicados especificamente à Fonoaudiologia. A literatura sinaliza iniciativas na Austrália^(32-34)^, no Chile^(35,36)^, nos Estados Unidos^(37)^, na África do Sul^(38)^, na Irlanda^(39)^, na Espanha^(40)^ e no Reino Unido^(4)^. Estas pesquisas apontam para a importância da autonomia do aprendiz em seu processo de aprendizagem e para a necessidade de propostas de formação de estudantes baseadas em estudos de casos, permitindo o desenvolvimento de habilidades de raciocínio clínico que se aproximem dos mecanismos utilizados por especialistas^(32,35)^. O pensamento crítico e o uso de instintos clínicos por parte dos profissionais são valorizados, sugerindo preponderância destes em relação às medidas de avaliação propriamente ditas^(38)^. Ressalta-se que a prática fonoaudiológica é centrada no paciente e no profissional e que o pensamento crítico é o componente da tomada de decisão, constituindo ferramenta para abordar os diferentes ingredientes e a natureza dinâmica da prática clínica^(39)^. A plataforma *web* proposta busca atender a estas recomendações, na medida em que: 1) lança luz sobre o raciocínio clínico de profissionais experientes; 2) utiliza-se de casos clínicos reais; 3) valoriza o pensamento crítico e a reflexão na aprendizagem e; 4) estimula a tomada de decisão fundamentada em ciência.

Os cenários clínicos - já em fase final de desenvolvimento - guiarão a experiência do usuário com a plataforma *web* por meio de perguntas e devolutivas oferecidas a partir das suas próprias tomadas de decisão, completando, assim, o guia de estudo da plataforma *Script* Voz. A ferramenta passará, então, por avaliações de usabilidade e de aceitação dos usuários, redefinição e determinação dos encargos, quando poderá ser disponibilizada ao público. Inicialmente alimentada com seis casos clínicos, a expectativa é de que seja gradativamente atualizada com novos cenários clínicos, configurando-se estratégia pedagógica cada vez mais diversificada na área de voz. Há ainda a previsão de tradução da plataforma *web* para a língua inglesa, buscando-se ampliar o seu alcance.

## CONCLUSÃO

A plataforma foi desenvolvida a partir da ferramenta Figma, utilizando-se a metodologia *Design Thinking* ^(12)^ como norteadora. A *Script* Voz será de livre acesso e seu protótipo de alta fidelidade encontra-se já hospedado no *site* da instituição de ensino que sedia a pesquisa (UFMG). Foram elaborados os guias de procedimento da plataforma, com orientações estabelecidas por consenso a partir de painel de especialistas. Tais guias têm por objetivo nortear os procedimentos para a coleta de dados, o estabelecimento dos diagnósticos de manifestação, do planejamento terapêutico e das condutas fonoaudiológicas voltadas para os casos clínicos que comporão a ferramenta. As perguntas e devolutivas que guiarão a experiência do usuário na plataforma *web* encontram-se em fase final de elaboração. A ferramenta usará casos clínicos reais e argumentos ancorados na literatura e no raciocínio clínico de profissionais experientes.
